# A National Survey to Assess the COVID-19 Vaccine-Related Conspiracy Beliefs, Acceptability, Preference, and Willingness to Pay among the General Population of Pakistan

**DOI:** 10.3390/vaccines9070720

**Published:** 2021-07-01

**Authors:** Muhammad Subhan Arshad, Iltaf Hussain, Tahir Mahmood, Khezar Hayat, Abdul Majeed, Imran Imran, Hamid Saeed, Muhammad Omer Iqbal, Muhammad Uzair, Anees ur Rehman, Waseem Ashraf, Areeba Usman, Shahzada Khurram Syed, Muqarrab Akbar, Muhammad Omer Chaudhry, Basit Ramzan, Muhammad Islam, Muhammad Usman Saleem, Waleed Shakeel, Iram Iqbal, Furqan Hashmi, Muhammad Fawad Rasool

**Affiliations:** 1 Department of Pharmacy Practice, Faculty of Pharmacy, Bahauddin Zakariya University, Multan 60800, Pakistan; m.subhan1995@gmail.com (M.S.A.); altaf9216@gmail.com (I.H.); abdulmajeed@bzu.edu.pk (A.M.); aneesurrehman@bzu.edu.pk (A.u.R.); 2 Department of Communication Studies, Bahauddin Zakariya University, Multan 60800, Pakistan; tahirmahmood@bzu.edu.pk; 3 Institute of Pharmaceutical Sciences, University of Veterinary and Animal Sciences, Lahore 54000, Pakistan; khezar.hayat@uvas.edu.pk; 4 Department of Pharmacy Administration and Clinical Pharmacy, School of Pharmacy, Xi’an Jiaotong University, Xi’an 710061, China; 5 Department of Pharmacology, Faculty of Pharmacy, Bahauddin Zakariya University, Multan 60800, Pakistan; imran.ch@bzu.edu.pk (I.I.); chishtiwaseem@yahoo.com (W.A.); waleed.shakeel@yahoo.com (W.S.); iramiqbal.bzu@gmail.com (I.I.); 6 Allama Iqbal Campus, University of the Punjab, University College of Pharmacy, Lahore 54000, Pakistan; hamid.pharmacy@pu.edu.pk (H.S.); islam.pharmacy@pu.edu.pk (M.I.); furqan.pharmacy@pu.edu.pk (F.H.); 7 Key Laboratory of Marine Drugs (Ministry of Education), Shandong Laboratory of Glycoscience and Glycoengineering, School of Medicine and Pharmacy, Ocean University of China Qingdao, Shandong 266003, China; oiqbal133@gmail.com; 8 Department of Pharmaceutical Chemistry, Faculty of Pharmacy, Bahauddin Zakariya University, Multan 60800, Pakistan; muhammaduzair@bzu.edu.pk; 9 Nishter Medical Hospital, Multan 59070, Pakistan; drausman@outlook.com; 10 Department of Basic Medical Sciences, School of Health Sciences, University of Management and Technology Lahore, Lahore 54770, Pakistan; shahzada.khurram@umt.edu.pk; 11 Department of Political Science, Bahauddin Zakariya University, Multan 60800, Pakistan; muqarrabakbar@bzu.edu.pk; 12 School of Economics, Bahauddin Zakariya University, Multan 60800, Pakistan; omer@bzu.edu.pk; 13 Al-Shifa Pharmacy, Multan 60650, Pakistan; basit.ramzan@hotmail.com; 14 Department of Biosciences, Faculty of Veterinary Sciences, Bahauddin Zakariya University, Multan 60800, Pakistan; usmansaleem@bzu.edu.pk

**Keywords:** COVID-19, COVID-19 vaccine, conspiracy theories, vaccine acceptance, vaccine hesitancy, general public

## Abstract

The current study aims to assess the beliefs of the general public in Pakistan towards conspiracy theories, acceptance, willingness to pay, and preference for the COVID-19 vaccine. A cross-sectional study was conducted through an online self-administered questionnaire during January 2021. The Chi-square test or Fisher exact test was utilized for statistical data analysis. A total of 2158 respondents completed the questionnaire, among them 1192 (55.2%) were male with 23.87 (SD: ±6.23) years as mean age. The conspiracy beliefs circulating regarding the COVID-19 vaccine were believed by 9.3% to 28.4% of the study participants. Among them, 1040 (48.2%) agreed to vaccinate on its availability while 934 (43.3%) reported the Chinese vaccine as their preference. The conspiracy beliefs of the participants were significantly associated with acceptance of the COVID-19 vaccine. The existence of conspiracy beliefs and low vaccine acceptance among the general population is a serious threat to successful COVID-19 vaccination.

## 1. Introduction

During the last year, the Coronavirus disease 2019 (COVID-19) pandemic has imposed a heavy disease burden all over the globe [1,2,3]. Globally, there were 102 million reported cases of COVID-19 till January 2021, with a death toll of 2.2 million. While in Pakistan, the number of these cases was 543,214 and the associated deaths were 11,623 [4]. The spread of this infectious disease has led the world to a humanitarian and economic crisis [5]. It is known that vaccination can play an important role in the prevention of such pandemics [6]. To control and prevent the spread of COVID-19, the researchers with the support of pharmaceutical industries have developed the COVID-19 vaccines in record time. Now, it is the responsibility of the international health agencies to design and implement policies for ensuring the successful administration of the COVID-19 vaccines to every corner of the world [7].

The acceptance of the vaccination by the general public is the most important factor for any successful immunization program [8], as the public is the ultimate decider of the success or failure of a vaccination program. The vaccine hesitancy among the public negatively affects its acceptance rate. It is well known that the conspiracies and religious beliefs of the public are associated with vaccine hesitancy. The studies during the 2009 influenza pandemic showed a low acceptance rate of vaccines in the general public of America, Australia, the United Kingdom (UK), Greece, and France (17–67%) [9,10,11,12]. In contrast with the developed countries, the refusal and hesitancy to accept vaccination are more common among the developing countries, as preventable diseases like polio are still prevalent in these countries [13,14]. In 2019, the World Health Organization (WHO) considered vaccine hesitancy as in the top ten threats to global health [15], therefore vaccine hesitancy may pose a major threat to COVID-19 vaccination, which may lead to the failure of its immunization program [16,17].

The anti-vaccination behavior of the general public has been reported previously with polio vaccination in Pakistan. This behavior was developed due to various conspiracy theories and superstitious religious beliefs of the general public regarding vaccination [18,19,20,21]. Amid the COVID-19 pandemic, the practice of non-pharmaceutical intervention like wearing a mask, using hand sanitizer, washing hands, and social distancing was less observed among the general population of Pakistan. This public behavior was thought to be associated with various conspiracies and religious beliefs [22,23]. Moreover, with the development of COVID-19 vaccines, various conspiracy theories have been promulgated around the globe. One of the most accepted theories concerning the COVID-19 vaccine is that of the installation of 5G chips in humans and its associated infertility [24,25,26,27]. Additionally, such conspiracy theories are circulating among the general public of Pakistan via social media [28]. In Pakistan, where vaccine hesitancy is the major barrier to curb vaccine-preventable diseases, such narratives may plant seeds of resistance against the COVID-19 vaccination program [20]. It is important to identify the source of myths in the current study. As has been previously reported, social media was the main source of spreading such myths [29]. This identification of the source of the conspiracy theories will assist the regulatory authorities in neutralizing the spread of such theories. Therefore, the next challenge that public health officials will face in developing countries like Pakistan is to overcome the superstitions and conspiracies associated with the COVID-19 vaccine. Therefore, to ensure an acceptable and effective COVID-19 vaccination program in Pakistan, it is important to investigate the prevalent conspiracies and the factors associated with anti-vaccination behavior.

The willingness to pay for vaccination is linked to vaccine acceptance. It has been reported in previous studies that vaccine price was significantly associated with vaccine acceptance [30,31]. Pakistan is a lower-middle-income country, where almost 35% of the population lives beneath the poverty line [32]. Therefore, it was considered important to assess the price range that the population is willing to pay for the COVID-19 vaccines. Moreover, vaccine preferences can play an important role in the adherence and acceptability of the vaccination program [33]. The preferences of the general public regarding COVID-19 vaccination will assist the health regulatory agencies in designing strategies that may ultimately increase vaccine acceptance.

This study was conducted with the primary objective to assess the beliefs of the general public of Pakistan regarding myths and conspiracy theories concerning the COVID-19 vaccine. The ultimate aim of the study was to evaluate the acceptance rate, willingness to pay, and preference for COVID-19 vaccines in the general public of Pakistan.

## 2. Materials and Methods

### 2.1. Study Design, Sampling, and Study Participants

A web-based cross-sectional study was conducted during January 2021 by utilizing a self-administrated online questionnaire, at the time when the drug regulatory authority of Pakistan (DRAP) was approving different COVID-19 vaccines for use in Pakistan. A convenient sampling technique was utilized to get maximum responses from the general population of Pakistan. Only residents of Pakistan who were aged more than or equal to 18 years were included in the study.

### 2.2. Ethical Considerations

The current study was conducted in accordance with the declaration of Helsinki. The ethical committee of the Department of Pharmacy Practice, Bahauddin Zakariya University, Multan (reference No. ACAD/PRAC/21-01) granted the formal approval for the study. Electronic consent was obtained from the participants with an introductory section of the survey detailing the purpose, nature of the survey, volunteer participation, and declaration of confidentiality. This paper has been reported as per the strengthening the reporting of observational studies in epidemiology (STROBE) guidelines [34] (Supplementary Information: Supplementary Table S1).

### 2.3. Survey Tool

The self-administered questionnaire was developed based on previous studies following the aim of the study [14,35,36,37]. The questionnaire was also translated into the national language of Pakistan (Urdu) for the convenience of the general public. The face validity of the study instrument and its Urdu version was confirmed by the relevant experts and their suggestion was incorporated in the final version of the instrument. Then, the improved and finalized questionnaire was used for the final data collection (Supplementary Information: Supplementary Table S2).

The final questionnaire comprised of four sections with a total of 21 items. The first section comprised of informed consent as an agreement to participate voluntarily and details about the study. The second section was based on demographic data, including questions regarding gender, age, marital status, education, occupation, profession, monthly income, province, and residential area. The third section was comprised of eight items assessing the beliefs and source of information about myths or conspiracy theories regarding the COVID-19 vaccine. The last section consisted of three items concerning the acceptance, willingness to pay, and preference regarding the COVID-19 vaccine. The categories related to willingness to pay for vaccination were created according to the estimated price of the COVID-19 vaccine in the international market then converting it to local currency [38,39]. The COVID-19 vaccines that were approved by the DRAP at the time of the study, were included in the study instrument for assessing participants’ vaccine preference [40]. The included COVID-19 vaccine brands were Pfizer-BioNTech, Oxford/AstraZeneca, and Sinopharm [41].

### 2.4. Data Collection

Google Forms (Google LLC, Mountain View, CA, USA) was used to design the online questionnaire. The questionnaire’s link was created and shared with the general public via social media platforms (Facebook, WhatsApp, and E-mail) based on the author’s approach. The questionnaire was distributed online via social media based on the author’s approach. The online survey link was shared with all the authors. Each author was responsible for sharing the online survey within a specific geographical area. To avoid incomplete responses, the answer to each question was mandatory for moving to the next section of the survey. All the items in the specific section were randomized to prevent bias. To avoid duplicate entries, a check was applied to get one response from one IP address. The incomplete questionnaires were excluded from the study. To avoid redundant answers from the non-serious participants, the responses completed in a time of less than 1-min were excluded from the final analysis. The checklist for reporting results of internet e-surveys (CHERRIES) guidelines was followed for online survey administration and data collection [42].

### 2.5. Statistical Analysis

The statistical package for the social sciences (SPSS) version 21.0 (IBM SPSS Statistics, New York, NY, USA) was used for the statistical analysis. The potential confounders were controlled after data collection by adding layer variables (as control) in the chi-square test. Descriptive statistics were used to present the demographic characteristics of the participants. The categorical variables were presented as frequency and percentage while continuous variables were presented as mean and standard deviation. The chi-squared (χ^2^) test or Fisher exact test was used to analyze the association between categorical variables as per need. The alpha value less than 0.05 was considered statistically significant. The Bonferroni correction was utilized for the repeated inferential statistics to control the type 1 error.

## 3. Results

Among the total 2246 participants, 2158 respondents completed the questionnaire (completeness rate: 96.0%). Among the total respondents, 1192 (55.2%) were male and 966 (44.8%) were female, with a mean age of 23.87 (SD: ± 6.23) years. The frequency of unmarried participants was 1764 (81.7%) and 1322 (61.3%) participants had a bachelor’s degree. Almost three-quarters of the participants were students with no monthly income (1550;71.8%), while more than half of the participants were from non-medical professions (1144;53.0%). The detail of the demographics of participants can be seen in Table 1.

**Figure 1:**
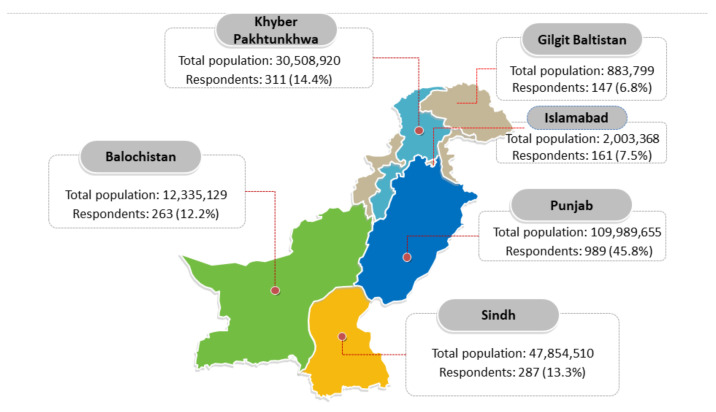
Provincial distribution of the respondents (source: Pakistan Bureau of Statistics) [43].

The majority of the respondents were from the province of Punjab (989;45.8%) followed by Khyber Pakhtunkhwa (311;14.4%) and Sindh (287;13.3%). The geographical distribution of the respondents is shown in Figure 1.

The belief of study participants varied with the type and nature of the conspiracy theory, as 410 (19.0%) respondents believe that the COVID-19 vaccine can kill people, while 200 (9.3%) believed that the COVID-19 vaccine contains 5G-nanochips to control people. Among the study participants, 285 (12.0%) believed that the vaccine can cause infertility, while 282 (13.1%) believed that the COVID-19 vaccine is designed to harm Muslims. Moreover, 354 (16.4%) respondents thought that COVID-19 and its vaccine is the propaganda of non-Muslims to rule the world. More than one-quarter of participants had concerns about the safety of the COVID-19 vaccine and believed that it could be harmful as it developed in a very short period (612;28.4%). The details of the conspiracy theory regarding the COVID-19 vaccine can be seen in Table 2.

**Figure 2:**
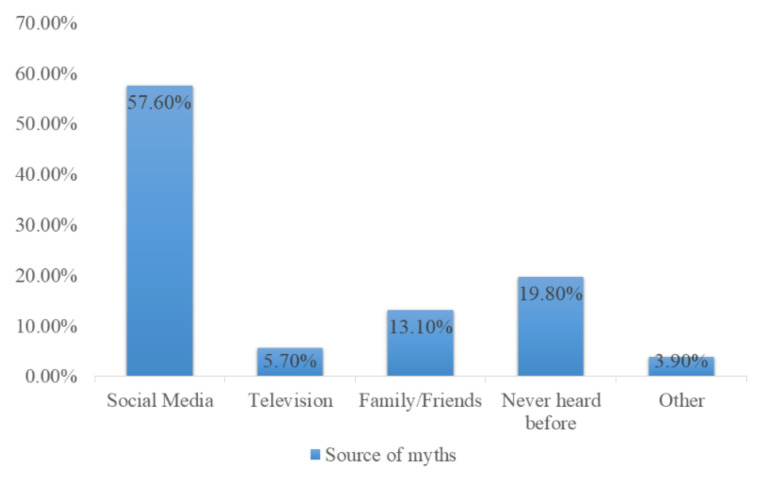
Sources of myths regarding the COVID-19 vaccine.

Furthermore, 1242 (57.6%) of participants mentioned social media as the major source of COVID-19 vaccine-related myths or conspiracy theories. The spreading sources of conspiracy theories related to the COVID-19 vaccine are given in Figure 2.

The acceptance of the COVID-19 vaccine was low as only half of the participants 1040 (48.2%) agreed to vaccinate themselves on its availability. Moreover, the majority of participants 934 (43.3%) showed their preference to vaccinate by the COVID-19 vaccine developed by the Sino Pharm (pharmaceutical company from China). Furthermore, half of the participants 1138 (52.7%) showed their willingness to pay less than 500 PKR (3.12 USD) for the COVID-19 vaccine. The details regarding willingness to pay and preferences regarding the COVID-19 vaccine are shown in Table 3.

**Figure 3:**
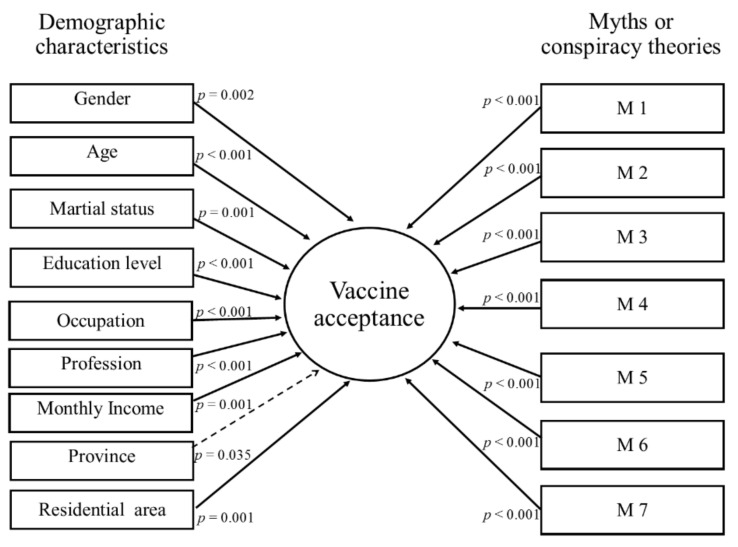
Association of COVID-19 vaccine acceptance with the demographic characteristics and myths. A solid line indicates a significant association (*p* < 0.05). A dashed line indicates a non-significant association (*p* ≥ 0.05). M 1—Do you believe that the COVID-19 vaccine has safety issues, which can kill people? M 2—Do you believe that the COVID-19 vaccine contains any 5G Nano-chips to control people? M 3—Do you believe that the COVID-19 vaccine could take away reproducibility (or cause infertility)? M 4—Do you believe that COVID-19 and its vaccine are created to control the world population? M 5—Do you believe that COVID-19 and its vaccine is designed to harm the Muslim nations? M 6—Do you believe that the COVID-19 vaccine can harm people’s health as this has been developed in a very short period? M 7—Do you believe COVID-19 and its vaccine are non-Muslims’ propaganda to rule the world?

Each conspiracy theory was significantly associated with the COVID-19 vaccine acceptance. Moreover, the demographic variables had a significant impact on the COVID-19 vaccine acceptance. The association between the demographics data of participants and their beliefs regarding myths about the COVID-19 vaccine can be seen in Supplementary Table S3 and Figure 3.

## 4. Discussion

To the best of our knowledge, the current study is the very first study that has assessed the beliefs of the general public of Pakistan regarding conspiracy theories about the COVID-19 vaccine and the source of such theories. Acceptance of the COVID-19 vaccine among the general population of Pakistan was also reported in relation to such myths. The most prominent belief of the general public in the current study was concerning the safety of the COVID-19 vaccine as it was developed and marketed in a very short time. Moreover, the majority of the participants considered that COVID-19 and its vaccine is propaganda of non-Muslims to rule the world. The most highlighted source of these conspiracy theories was social media platforms.

The conspiracy theories promulgated about the COVID-19 vaccine are being believed by 9.3% to 28.4% of the respondents in the current study. The most common myths that the COVID-19 vaccine can cause infertility and has 5G-nanochips were believed by 12.0% and 9.3% of the respondents. This number was comparatively low with a reported study from the Arab countries, where the frequency of believers regarding these myths were 27.7% and 23.4%, respectively [36]. Such conspiracy theories were also reported during the polio vaccination program in Pakistan and were considered as a major contributing factor to the anti-vaccination behavior of the public. The previous workup by the health ministry of Pakistan to eradicate and overcome the polio-vaccine-related myths could be the reason behind fewer believers of these myths in Pakistan as compared to the Arab world [14,44,45,46,47,48]. Moreover, the second reason could be that the majority of participants of the survey are university graduates who may have already good knowledge about COVID-19 vaccines. The public belief in such conspiracy theories is usually related to their mistrust in government, vaccine manufacturing companies, and healthcare professionals [49,50]. COVID-19 has already been linked with conspiracy theories from the beginning [51,52,53], while similar conspiracy theories regarding the COVID-19 vaccine are now prevalent in the general public and this can ultimately lead to vaccine hesitancy or refusal as a hurdle to control this pandemic [27,54,55].

The circulating and spreading sources of conspiracy theories regarding the COVID-19 vaccine were also addressed in this study, in which the majority of participants reported social media as a prominent source of the spread of such theories (57.55%). In the modern world, social media platforms are the major source of spread of conspiracy theories, especially during the COVID-19 pandemic [52,53,54]. The misleading information regarding the COVID-19 vaccine can be neutralized by implementing national public awareness campaigns at the government level to enhance vaccine acceptance among the public, which could be a tool to end this devastating pandemic.

In the current study, only 48.2% of respondents agreed to vaccinate against COVID-19, which indicated a low acceptance rate among the general public. A study from the Arab countries assessing the acceptance rate of the COVID-19 vaccine reported that only 29.4% of respondents were willing to vaccinate [17,56]. The differences in vaccine acceptance rates between the Arab countries and Pakistan can be related to the low frequency of believers of myths regarding COVID-19 and its vaccine seen in our study [28,56,57]. It has been reported that the vaccine acceptance rate in the United States (US) general population was 57.6% to 68.6%, which is high in comparison to our study [58,59]. A survey from 19 countries around the globe reported an acceptance rate, ranging from 55% in the Russian population to 90% in the Chinese population [60]. An acceptance rate higher than 90% was reported from earlier studies from Indonesia and China [61,62]. The low literacy rate and various religious factors may account for the low vaccine acceptance rate among the general population of Pakistan [61]. The higher vaccine acceptance rates in developed countries are related to low vaccine hesitancy and less belief in conspiracies regarding COVID-19 and its vaccines among their residents [63,64,65].

It has been observed in the current study that the majority of participants preferred the Chinese vaccine (43.3%) and half of the participants showed their willingness to pay less than 500 PKR (3.12 USD) (52.7%). The previous vaccination programs (polio) in Pakistan have been associated with different conspiracies, as people believed that these vaccines were developed by the western world to target Muslim nations, and the presence of such conspiracy beliefs could be the probable reason for low preferences for vaccines developed in western countries [14]. Pakistan is a lower-middle-income country and its economy has been affected by the COVID-19 pandemic, which has resulted in an increase in unemployment and poverty [66], which can be the reason that the general public wants to pay the lowest possible price for the vaccine.

There are some limitations and strengths of the current study. Firstly, it was based on a self-administered web-based questionnaire, so only those people who had internet access participated in this study. Therefore, we cannot generalize the results of this study to the whole population. Moreover, the time when the current study was conducted could be regarded as its strength, as this study was conducted during a time when DRAP was granting approvals for the different COVID-19 vaccines to be used in Pakistan, which makes this time conducive for the spread of conspiracy theories against this vaccine. The inclusion of participants from all provinces of Pakistan makes the presented study a nationwide survey and it can be considered as its strength.

## 5. Conclusions

The results of this study revealed the existence of conspiracy beliefs regarding the COVID-19 vaccine with a low acceptance rate among the general public of Pakistan. These beliefs are posing a major threat to the upcoming immunization program for the control of COVID-19 in Pakistan.

### Recommendations

The health administration and the policymakers should take immediate actions to neutralize this misleading information through evidence-based public education regarding the safety and efficacy of vaccines. The present study also suggested that social media was mainly responsible for the spread of conspiracy beliefs among the general population. It is suggested that the regulatory authorities should take concrete steps in countering the spread of such conspiracy beliefs. This can be done by advocating vaccine safety and disease prevention messages at the national level using electronic and social media platforms.

## Figures and Tables

**Table 1:** Demographic characteristics of study participants (*n* = 2158).

Characteristics		Frequency or Mean	Percentage or ± SD
Gender	Male	1192	55.2%
	Female	966	44.8%
Age (years)		23.87	6.23
Age groups	18–30 years	1984	91.9%
	31–50 Years	150	7.0%
	>50 years	24	1.1%
Marital status	Unmarried	1764	81.7%
	Married	384	17.8%
	Divorced	10	0.5%
Education level	Primary	12	0.6%
	Secondary	14	0.6%
	Intermediate	206	9.5%
	Bachelor	1322	61.3%
	Master or above	604	28.0%
Occupation	Student	1550	71.8%
	Govt. Employee	134	6.2%
	Non-Govt. Employee	196	9.1%
	Businessman	60	2.8%
	Unemployment	132	6.1%
	Housewife	86	4.0%
Profession	Not related to health	1144	53.0%
	Health related	1014	47.0%
Monthly income (PKR)	<20,000	156	7.2%
	20,000 to 40,000	178	8.2%
	40,001 to 60,000	102	4.7%
	>60,000	172	8.0%
	No income	1550	71.8%
Province	Punjab	989	45.8%
	Sindh	287	13.3%
	KPK	311	14.4%
	Baluchistan	263	12.2%
	Gilgit-Baltistan	147	6.8%
	Capital Islamabad	161	7.5%
Residence	Rural	580	26.9%
	Urban	1578	73.1%

**Table 2:** Beliefs of participants towards myths or conspiracy theories regarding the COVID-19 vaccine.

Myths or Conspiracy Theories		No *n* (%)	Maybe *n* (%)	Yes *n* (%)
M 1	Do you believe that the COVID-19 vaccine has safety issues, which can kill people?	732 (33.9%)	1016 (47.1%)	410 (19.0%)
M 2	Do you believe that the COVID-19 vaccine contains any 5G Nano-chips to control people?	1232 (57.1%)	726 (33.6%)	200 (9.3%)
M 3	Do you believe that the COVID-19 vaccine could take away reproducibility (or cause infertility)?	1110 (51.4%)	790 (36.6%)	258 (12.0%)
M 4	Do you believe that COVID-19 and its vaccine are created to control the world population?	1154 (53.5%)	606 (28.1%)	398 (18.4%)
M 5	Do you believe that COVID-19 and its vaccine is designed to harm the Muslim nations?	1424 (66.0%)	452 (20.9%)	282 (13.1%)
M 6	Do you believe that the COVID-19 vaccine can harm people’s health as this has been developed in a very short period?	636 (29.5%)	910 (42.2%)	612 (28.4%)
M 7	Do you believe COVID-19 and its vaccine are non-Muslims’ propaganda to rule the world?	1232 (57.1%)	572 (26.5%)	354 (16.4%)

**Table 3:** The acceptance, willingness to pay, and preference of participants regarding the COVID-19 vaccine.

		Frequency	Percentage
Vaccine Acceptance	No	556	25.8%
	Maybe	562	26.0%
	Yes	1040	48.2%
Willingness to pay	<500 PKR (3.12 USD)	1138	52.7%
	500 to 1001 PKR (3.12 to 6.25 USD)	654	30.3%
	1001 to 2000 PKR (6.26 to 12.50 USD)	146	6.8%
	2001 to 3000 PKR (12.51 to 18.75 USD)	76	3.5%
	>3000 PKR (18.75 USD)	144	6.7%
Preference	American Vaccine (Pfizer-biotech vaccine)	630	29.2%
	European vaccine (Oxford-AstraZeneca vaccine)	594	27.5%
	Chinese vaccine (Sino pharm)	934	43.3%

## Data Availability

All the data that was used for the analysis are available with the corresponding author (fawadrasool@bzu.edu.pk) and can be provided on a suitable request.

## Supplementary Materials

The following are available online at https://www.mdpi.com/article/10.3390/vaccines9070720/s1, Supplementary Table S1: STROBE Statement. Supplementary Table S2: Survey Tool. Supplementary Table S3: Association between the demographic data of the participants and their beliefs regarding myths about the COVID-19 vaccine.
