# Supplementary material for: A National Survey to Assess the COVID-19 Vaccine-Related Conspiracy Beliefs, Acceptability, Preference, and Willingness to Pay among the General Population of Pakistan
Source: Vaccines (Basel). 2021 Jul 1;9(7):720. doi: 10.3390/vaccines9070720 (PMC8310108; doi:10.3390/vaccines9070720)
Supplement: Supplementary file 1 [file vaccines-09-00720-s001.zip › vaccines-1278660-supplementary.pdf]

Supplementary Information

Supplementary Table S1

STROBE Statement—Checklist of items that should be included in reports of ***cross-sectional studies.***

	Item No	Recommendation
**Title and abstract**	1	(*a*) Indicate the study's design with a commonly used term in the title or the abstract (yes)
		(*b*) Provide in the abstract an informative and balanced summary of what was done and what was found (yes)
Background/rationale	2	Explain the scientific background and rationale for the investigation being reported (yes)
Objectives	3	State specific objectives, including any prespecified hypotheses (yes)
Study design	4	Present key elements of study design early in the paper (yes)
Setting	5	Describe the setting, locations, and relevant dates, including periods of recruitment, exposure, follow-up, and data collection (yes)
Participants	6	(*a*) Give the eligibility criteria, and the sources and methods of selection of participants (yes)
Variables	7	Clearly define all outcomes, exposures, predictors, potential confounders, and effect modifiers. Give diagnostic criteria, if applicable (yes)
Data sources/ measurement	8*	For each variable of interest, give sources of data and details of methods of assessment (measurement). Describe comparability of assessment methods if there is more than one group
Bias	9	Describe any efforts to address potential sources of bias (yes)
Study size	10	Explain how the study size was arrived at (yes)
Quantitative variables	11	Explain how quantitative variables were handled in the analyses. If applicable, describe which groupings were chosen and why (No)
Statistical methods	12	(*a*) Describe all statistical methods, including those used to control for confounding (yes)
		(*b*) Describe any methods used to examine subgroups and interactions (yes)
		(*c*) Explain how missing data were addressed (No)
		(*d*) If applicable, describe analytical methods taking account of sampling strategy (No)
		(*e*) Describe any sensitivity analyses (No)
Participants	13*	(a) Report numbers of individuals at each stage of study—eg numbers potentially eligible, examined for eligibility, confirmed eligible, included in the study, completing follow-up, and analysed (yes)
		(b) Give reasons for non-participation at each stage (No)
		(c) Consider use of a flow diagram (No)
Descriptive data	14*	(a) Give characteristics of study participants (eg demographic, clinical, social) and information on exposures and potential confounders (yes)
		(b) Indicate number of participants with missing data for each variable of interest (No)
Outcome data	15*	Report numbers of outcome events or summary measures (yes)

Main results	16	(*a*) Give unadjusted estimates and, if applicable, confounder-adjusted estimates and their precision (eg, 95% confidence interval). Make clear which confounders were adjusted for and why they were included
		(*b*) Report category boundaries when continuous variables were categorized (yes)
		(*c*) If relevant, consider translating estimates of relative risk into absolute risk for a meaningful time period (No)
Other analyses	17	Report other analyses done—eg analyses of subgroups and interactions, and sensitivity analyses (yes)
Key results	18	Summarise key results with reference to study objectives (yes)
Limitations	19	Discuss limitations of the study, taking into account sources of potential bias or imprecision. Discuss both direction and magnitude of any potential bias (yes)
Interpretation	20	Give a cautious overall interpretation of results considering objectives, limitations, multiplicity of analyses, results from similar studies, and other relevant evidence (yes)
Generalisability	21	Discuss the generalisability (external validity) of the study results (yes)
Funding	22	Give the source of funding and the role of the funders for the present study and, if applicable, for the original study on which the present article is based (yes)

*Give information separately for exposed and unexposed groups.

Note: An Explanation and Elaboration article discusses each checklist item and gives methodological background and published examples of transparent reporting. The STROBE checklist is best used in conjunction with this article (freely available on the Web sites of PLoS Medicine at http://www.plosmedicine.org/, Annals of Internal Medicine at http://www.annals.org/, and Epidemiology at http://www.epidem.com/). Information on the STROBE Initiative is available at www.strobe-statement.org.

Supplementary Table S2. Survey Tool

<table>
<tr><td colspan="2">A national survey to assess the COVID-19 vaccine-related conspiracy beliefs, acceptability, preference, and willingness to pay among the general population of Pakistan.
ویکسین سے متعلق سازش کے عقائد ، قبولیت ، ترجیح ، اور پاکستان کی COVID-19 ایک قومی سروے جس میں عام آبادی کے درمیان ادائیگی کے لئے آمادگی کا جائزہ لیا گیا ہے</td></tr>
<tr><td colspan="2">Section 1. Informed Consent
سیکشن 1. باخبر رضامندی

Assalam o Alaikum! We are from the Department of Pharmacy Practice, BZU Multan. We are working on a project concerned with COVID-19 Vaccine myths, acceptability, preference, and willingness to pay; a cross-sectional study among the general public of Pakistan, in which you could participate. There are many conspiracy theories and myths spreading about the COVID-19 vaccine, which could cause hindrance in vaccination programs. The purpose of the study to assess the general public's beliefs about myths regarding the COVID-19 vaccine and how they will respond to vaccination & willingness to pay for it. The data from this web-based study will be helpful to neutralize such conspiracy theories about the COVID-19 vaccine to increase its acceptability in Pakistan. This survey doesn't contain any identifying information about you. Therefore, your responses will remain anonymous. No one will be able to identify you or your answers, and no one will know whether or not you participated in the study.

السلام علیکم!ھمارا تعلق شعبہ فارمیسی پریکٹس، جامعۂ بہاءالدین زکریا، ملتان سے ہے۔ ہم پاکستان کی عام عوام میں کوویڈ-19 ویکسین کےبارےمیں پائی جانے والی افواہوں پر لوگوں کا یقین، ویکسین لگوانے اور خریدنیں کے بارے میں لوگوں کی راضامندی اور ترجیح پر مبنی ایک سروےکر رھے ھیں ، جس میں آپ حصہ لے سکتے ہیں۔ کوویڈ-19 ویکسین کے بارے میں بہت سی سازشی نظریات اور افواہیں پھیل رہی ہیں جوویکسینیشن پروگراموں میں رکاوٹ کا سبب بن سکتی ہیں۔ اس سروے کا مقصد کوویڈ-19 ویکسین سے متعلق خرافات کے بارے میں عام لوگوں کےاعتقادات کا جائزہ لینا ہے۔ اس ویب پر مبنی مطالعے کا ڈیٹا پاکستان میں کوویڈ-19 ویکسین کے بارے میں ایسے سازشی نظریات کو بے اثر کرنےمیں اور قبولیت بڑھانے کے لئے مددگار ثابت ہوگا۔ اس سروے میں آپ کی شناخت کوظاھر کرنے والی کسئی معلومات کے بارے میں نہیں پوچھاگیا۔لہذا ، آپ کے جوابات گمنام رہیں گے۔ کوئی بھی آپ یا آپ کے جوابات کی شناخت نہیں کر سکے گا ، اور کسی کو یہ معلوم نہیں ہوگا کہ آپ نےمطالعہ میں حصہ لیا ہے یا نہیں۔</td></tr>
<tr><td>1. Do you agree to participate in this survey?
1. کیا آپ اس سروے میں حصہ لینےکے لئے راضی ہیں؟</td><td>☐ (جی ہاں) Yes
☐ (نہیں) No</td></tr>
</table>

**Section 2. Demographics.**<br>**سیکشن 2. آبادیات.**	
2. What is your gender?<br>2. آپ کی جنس کیا ہے؟	Male (مرد)<br>)عورتFemale (
3. What is your age? (Years)<br>3. آپ کی عمر کیا ہے؟ (سالوں میں)	Years(سال) ________
4. What is your marital status?<br>4. آپ کی ازدواجی حیثیت کیا ہے؟	(غیر شادی شدہ)Unmarried<br>(شادی شدہ)Married<br>)طلاق شدہDivorced(
5. What is your education?<br>5. آپ کی تعلیم کیا ہے؟	) (پرائمری)Primary (1-5<br>) (سیکنڈری)Secondary (6-10<br>) (انٹرمیڈیٹ)Intermediate (11-12<br>) (بیچلر)Bachelor (12-16<br>(ماسٹر یا اس سے اوپر)Master or above
6. To which profession do you belong?<br>6. آپ کس شعبے سے تعلق رکھتے ہیں؟	Health-related (MBBS, BDS, Nursing, Pharmacy & DPT etc.) (صحت سے متعلق)<br>Not related to health (Other than mention above)<br>)صحت سے غیر متعلق(
7. What is Your monthly income? (PKR)<br>7. آپ کی ماہانہ آمدنی کتنی ہے؟ (روپے)	<20,000<br>20,000 to 40,000<br>40,001 to 60,000<br>>60,000<br>) No income(کوئی آمدنی نہیں
8. To which province do you belong?<br>8. آپ کس صوبے سے تعلق رکھتے ہیں؟	پنجاب) Punjab (<br>)سندھ Sindh (<br>) خیبر پختونخوا Khyber Pakhtunkhwa (KPK)(<br>)بلوچستان Baluchistan (<br>)گلگت بلتستان Gilgit-Baltistan (<br>)دارالحکومت اسلام آباد Capital Islamabad (
9. What is your residential area?<br>9. آپ کا رہائشی علاقہ کیا ہے؟	)دیہی Rural (<br>)شہری Urban (
**Section 3. Myths/ conspiracy theories about COVID-19 Vaccine**<br>**ویکسین کے بارے میں خرافات / سازش کے نظریات19سیکشن 3. کوویڈ-**	

10. Do you believe that the COVID-19 vaccine has safety issues, which can kill people?<br>ویکسین میں حفاظتی مسائل ہیں ، جو کہ 19. کیا آپ کولگتا ہےکہ کوویڈ-10<br>لوگوں کی موت کی واجہ بن سکتے ہیں؟	Yes<br>)جی ہاں(	Maybe<br>(شاید)	No<br>(نہیں)
11. Do you believe that the COVID-19 vaccine contains any 5G Nano-chips to control people?<br>ویکسین میں لوگوں کو قابو میں کرنے 1119. کیا آپ کو لگتا ہے کہ کوویڈ-<br>جی نینو چپس موجود ہیں؟5کیلے کوئی	Yes<br>)جی ہاں(	Maybe<br>(شاید)	No<br>(نہیں)
12. Do you believe that the COVID-19 vaccine could take away reproducibility (or cause infertility)?<br>ویکسین آپکی تولیدی صلاحیت ختم کر 1219. کیا آپ کولگتا ہے کہ کوویڈ-<br>سکتی ہے(یا بانجھ پن کی وجہ بن سکتی ہے) ؟	Yes<br>)جی ہاں(	Maybe<br>(شاید)	No<br>(نہیں)
13. Do you believe that COVID-19 and its vaccine are created to control the world population?<br>اور اس کی ویکسین دنیا کی آبادی کو 1319. کیا آپ کو لگتا ہے کہ کوویڈ-<br>کنٹرول کرنے کے لئے بنائی گئی ہے؟	Yes<br>)جی ہاں(	Maybe<br>(شاید)	No<br>(نہیں)
14. Do you believe that COVID-19 and its vaccine is designed to harm the Muslim nations?<br>اور اسکی ویکسین مسلم قوموں کو 1419. کیا اپ کو لگتا ہے کہ کوویڈ-<br>نقصان پہنچانے کے لیے بنائی گئی ہیں؟	Yes<br>)جی ہاں(	Maybe<br>(شاید)	No<br>(نہیں)
15. Do you believe that the COVID-19 vaccine can harm people's health as this has been developed in a very short period?<br>ویکسین لوگوں کی صحت کو نقصان 1519. کیا آپ کو لگتا ہے کہ کوویڈ-<br>پہنچا سکتی ہے کیوں کہ یہ بہت ہی مختصر عرصے میں تیار کی گی ہے؟	Yes<br>)جی ہاں(	Maybe<br>(شاید)	No<br>(نہیں)
16. Do you believe COVID-19 and its vaccine are non-Muslims' propaganda to rule the world?<br>اور اس کی ویکسین دنیا پر حکمرانی 1619. کیا آپ کو لگتا ہے کہ کوویڈ -<br>کے لئے غیر مسلموں کا پروپیگنڈا(سازش) ہے؟	Yes<br>)جی ہاں(	Maybe<br>(شاید)	No<br>(نہیں)
18. From where you have heard about these Myths/ Conspiracy theories?<br>18. آپ نے ان خرافات / سازش کے نظریات کے بارے<br>میں کہاں سے سنا ہے؟	Social media (WhatsApp, Facebook, etc.)) سوشل میڈیا(<br>)اخبار Newspaper(<br>)ٹیلی ویژن Television (<br>)فیملی/دوست(Family/Friends (<br>)پہلے کبھی نہیں سنا Never heard before (		

Section 4. the acceptance, willingness to pay, and preference regarding vaccine of COVID-19

سیکشن 4. کوویڈ 19 کے ویکسین کی منظوری ، ادا کرنے پر آمادگی ، اور ترجیح

19. Would you vaccinate yourself on its availability of the COVID-19 vaccine in Pakistan?<br>ویکسین کی 1919. کیا آپ پاکستان میں کوویڈ-<br>دستیابی پر اپنے آپ کو ویکسین لگوایں گے؟	(جی ہاں)Yes<br>(نہیں)No<br>(شاید)Maybe
20. What is the maximum amount you are willing to pay the COVID-19 vaccine per dose for your own vaccination? (PKR)<br>2019. آپ خود کوویکسین لگوانےکیلئے کوویڈ-<br>ویکسین فی خوراک زیادہ سے زیادہ کتنی رقم ادا<br>کرنےکیلے راضی ہیں؟(روپے)	500><br>500to 1000<br>1001to 2000<br>20013000 to<br>3000<
21. Which COVID-19 vaccine do you prefer for yourself?<br>ویکسین لگوانا پسند 2119. آپ خود کو کون سی کوویڈ-<br>کریں گے؟	American Vaccine (Pfizer-biotech vaccine)<br>(امریکی ویکسین)<br>European vaccine (Oxford-AstraZeneca vaccine)(یورپی ویکسین)<br>Chinese vaccine (Sino pharm)(چینیز ویکسین)

Supplementary Table S3. Association between the demographic data of the participants and their beliefs regarding myths about the COVID-19 vaccine.

		M1. Do you believe that the COVID-19 vaccine has safety issues, which can kill people?						P-value
		No		Maybe		Yes		
		N	%	N	%	N	%	
Gender	Male	394	53.8%	548	53.9%	250	61.0%	0.034
	Female	338	46.2%	468	46.1%	160	39.0%	
Age Groups	18-30 years	660	90.2%	932	91.7%	392	95.6%	<0.001
	31-50 Years	56	7.7%	80	7.9%	14	3.4%	
	>50 years	16	2.2%	4	0.4%	4	1.0%	
Marital status	Unmarried	598	81.7%	834	82.1%	332	81.0%	0.014
	Married	134	18.3%	178	17.5%	72	17.6%	
	Divorced	0	0.0%	4	0.4%	6	1.5%	
Education	Primary	6	0.8%	2	0.2%	4	1.0%	0.677
	Secondary	4	0.5%	8	0.8%	2	0.5%	
	Intermediate	66	9.0%	102	10.0%	38	9.3%	
	Bachelor	448	61.2%	622	61.2%	252	61.5%	
	Master or above	208	28.4%	282	27.8%	114	27.8%	
Occupation	Student	482	65.8%	766	75.4%	302	73.7%	0.002
	Govt. Employee	54	7.4%	58	5.7%	22	5.4%	
	Non-Govt. Employee	92	12.6%	76	7.5%	28	6.8%	
	Businessman	24	3.3%	22	2.2%	14	3.4%	
	Unemployment	50	6.8%	56	5.5%	26	6.3%	
	Housewife	30	4.1%	38	3.7%	18	4.4%	
Profession	Not related to health	350	47.8%	564	55.5%	230	56.1%	0.002
	Health related	382	52.2%	452	44.5%	180	43.9%	
Monthly income	<20,000	46	6.3%	72	7.1%	38	9.3%	<0.001
	20,000 to 40,000	78	10.7%	68	6.7%	32	7.8%	
	40,001 to 60,000	48	6.6%	40	3.9%	14	3.4%	
	>60,000	70	9.6%	64	6.3%	38	9.3%	
	No income	490	66.9%	772	76.0%	288	70.2%	
Province	Punjab	337	46.0%	460	45.3%	192	46.8%	0.224
	Sindh	96	13.1%	132	13.0%	59	14.4%	
	KPK	115	15.7%	154	15.2%	42	10.2%	
	Baluchistan	76	10.4%	128	12.6%	59	14.4%	
	Gilgit-Baltistan	51	7.0%	73	7.2%	23	5.6%	
	Capital Islamabad	57	7.8%	69	6.8%	35	8.5%	
Residence	Rural	202	27.6%	254	25.0%	124	30.2%	0.112
	Urban	530	72.4%	762	75.0%	286	69.8%	

		M2. Do you believe that the COVID-19 vaccine contains any 5G Nano-chips to control people?						P-value
		No		Maybe		Yes		
		N	%	N	%	N	%	
Gender	Male	676	54.9%	402	55.4%	114	57.0%	0.851
	Female	556	45.1%	324	44.6%	86	43.0%	
Age Groups	18-30 years	1120	90.9%	676	93.1%	188	94.0%	0.079
	31-50 Years	96	7.8%	46	6.3%	8	4.0%	
	>50 years	16	1.3%	4	0.6%	4	2.0%	
Marital status	Unmarried	1000	81.2%	606	83.5%	158	79.0%	<0.001
	Married	232	18.8%	116	16.0%	36	18.0%	
	Divorced	0	0.0%	4	0.6%	6	3.0%	
Education	Primary	8	0.6%	2	0.3%	2	1.0%	0.023
	Secondary	4	0.3%	6	0.8%	4	2.0%	
	Intermediate	108	8.8%	70	9.6%	28	14.0%	
	Bachelor	752	61.0%	458	63.1%	112	56.0%	
	Master or above	360	29.2%	190	26.2%	54	27.0%	
Occupation	Student	842	68.3%	562	77.4%	146	73.0%	<0.001
	Govt. Employee	90	7.3%	28	3.9%	16	8.0%	
	Non-Govt. Employee	136	11.0%	48	6.6%	12	6.0%	
	Businessman	36	2.9%	14	1.9%	10	5.0%	
	Unemployment	86	7.0%	40	5.5%	6	3.0%	
	Housewife	42	3.4%	34	4.7%	10	5.0%	
Profession	Not related to health	578	46.9%	456	62.8%	110	55.0%	<0.001
	Health related	654	53.1%	270	37.2%	90	45.0%	
Monthly income	<20,000	98	8.0%	48	6.6%	10	5.0%	<0.001
	20,000 to 40,000	112	9.1%	50	6.9%	16	8.0%	
	40,001 to 60,000	64	5.2%	24	3.3%	14	7.0%	
	>60,000	106	8.6%	38	5.2%	28	14.0%	
	No income	852	69.2%	566	78.0%	132	66.0%	
Province	Punjab	578	46.9%	327	45.0%	84	42.0%	0.057
	Sindh	169	13.7%	83	11.4%	35	17.5%	
	KPK	170	13.8%	110	15.2%	31	15.5%	
	Baluchistan	134	10.9%	110	15.2%	19	9.5%	
	Gilgit-Baltistan	81	6.6%	48	6.6%	18	9.0%	
	Capital Islamabad	100	8.1%	48	6.6%	13	6.5%	
Residence	Rural	296	24.0%	232	32.0%	52	26.0%	0.001
	Urban	936	76.0%	494	68.0%	148	74.0%	

		M3. Do you believe that the COVID-19 vaccine could take away reproducibility (or cause infertility)?						P-value
		No		Maybe		Yes		
		N	%	N	%	N	%	
Gender	Male	592	53.3%	432	54.7%	168	65.1%	0.003
	Female	518	46.7%	358	45.3%	90	34.9%	
Age Groups	18-30 years	1006	90.6%	734	92.9%	244	94.6%	0.044
	31-50 Years	88	7.9%	52	6.6%	10	3.9%	
	>50 years	16	1.4%	4	0.5%	4	1.6%	
Marital status	Unmarried	904	81.4%	642	81.3%	218	84.5%	0.024
	Married	204	18.4%	144	18.2%	36	14.0%	
	Divorced	2	0.2%	4	0.5%	4	1.6%	
Education	Primary	8	0.7%	2	0.3%	2	0.8%	0.334
	Secondary	8	0.7%	4	0.5%	2	0.8%	
	Intermediate	102	9.2%	74	9.4%	30	11.6%	
	Bachelor	658	59.3%	504	63.8%	160	62.0%	
	Master or above	334	30.1%	206	26.1%	64	24.8%	
Occupation	Student	760	68.5%	584	73.9%	206	79.8%	<0.001
	Govt. Employee	78	7.0%	44	5.6%	12	4.7%	
	Non-Govt. Employee	126	11.4%	60	7.6%	10	3.9%	
	Businessman	32	2.9%	16	2.0%	12	4.7%	
	Unemployment	78	7.0%	52	6.6%	2	0.8%	
	Housewife	36	3.2%	34	4.3%	16	6.2%	
Profession	Not related to health	530	47.7%	468	59.2%	146	56.6%	<0.001
	Health related	580	52.3%	322	40.8%	112	43.4%	
Monthly income	<20,000	86	7.7%	54	6.8%	16	6.2%	0.002
	20,000 to 40,000	96	8.6%	58	7.3%	24	9.3%	
	40,001 to 60,000	48	4.3%	32	4.1%	22	8.5%	
	>60,000	106	9.5%	44	5.6%	22	8.5%	
	No income	774	69.7%	602	76.2%	174	67.4%	
Province	Punjab	517	46.6%	361	45.7%	111	43.0%	0.047
	Sindh	142	12.8%	101	12.8%	44	17.1%	
	KPK	165	14.9%	118	14.9%	28	10.9%	
	Baluchistan	117	10.5%	110	13.9%	36	14.0%	
	Gilgit-Baltistan	88	7.9%	46	5.8%	13	5.0%	
	Capital Islamabad	81	7.3%	54	6.8%	26	10.1%	
Residence	Rural	278	25.0%	218	27.6%	84	32.6%	0.042
	Urban	832	75.0%	572	72.4%	174	67.4%	

		M4. Do you believe that COVID-19 and its vaccine are created to control the world population?						P-value
		No		Maybe		Yes		
		N	%	N	%	N	%	
Gender	Male	622	53.9%	348	57.4%	222	55.8%	0.358
	Female	532	46.1%	258	42.6%	176	44.2%	
Age Groups	18-30 years	1054	91.3%	566	93.4%	364	91.5%	0.016
	31-50 Years	88	7.6%	38	6.3%	24	6.0%	
	>50 years	12	1.0%	2	0.3%	10	2.5%	
Marital status	Unmarried	936	81.1%	500	82.5%	328	82.4%	0.011
	Married	216	18.7%	104	17.2%	64	16.1%	
	Divorced	2	0.2%	2	0.3%	6	1.5%	
Education	Primary	2	0.2%	2	0.3%	8	2.0%	<0.001
	Secondary	4	0.3%	4	0.7%	6	1.5%	
	Intermediate	114	9.9%	42	6.9%	50	12.6%	
	Bachelor	684	59.3%	402	66.3%	236	59.3%	
	Master or above	350	30.3%	156	25.7%	98	24.6%	
Occupation	Student	796	69.0%	452	74.6%	302	75.9%	<0.001
	Govt. Employee	72	6.2%	40	6.6%	22	5.5%	
	Non-Govt. Employee	130	11.3%	50	8.3%	16	4.0%	
	Businessman	40	3.5%	6	1.0%	14	3.5%	
	Unemployment	76	6.6%	40	6.6%	16	4.0%	
	Housewife	40	3.5%	18	3.0%	28	7.0%	
Profession	Not related to health	518	44.9%	380	62.7%	246	61.8%	<0.001
	Health related	636	55.1%	226	37.3%	152	38.2%	
Monthly income	<20,000	86	7.5%	44	7.3%	26	6.5%	0.024
	20,000 to 40,000	106	9.2%	46	7.6%	26	6.5%	
	40,001 to 60,000	58	5.0%	18	3.0%	26	6.5%	
	>60,000	104	9.0%	36	5.9%	32	8.0%	
	No income	800	69.3%	462	76.2%	288	72.4%	
Province	Punjab	546	47.3%	272	44.9%	171	43.0%	0.156
	Sindh	160	13.9%	68	11.2%	59	14.8%	
	KPK	153	13.3%	105	17.3%	53	13.3%	
	Baluchistan	127	11.0%	84	13.9%	52	13.1%	
	Gilgit-Baltistan	78	6.8%	39	6.4%	30	7.5%	
	Capital Islamabad	90	7.8%	38	6.3%	33	8.3%	
Residence	Rural	296	25.6%	186	30.7%	98	24.6%	0.041
	Urban	858	74.4%	420	69.3%	300	75.4%	

		M5. Do you believe that COVID-19 and its vaccine is designed to harm the Muslim nations?						P-value
		No		Maybe		Yes		
		N	%	N	%	N	%	
Gender	Male	760	53.4%	256	56.6%	176	62.4%	0.016
	Female	664	46.6%	196	43.4%	106	37.6%	
Age Groups	18-30 years	1308	91.9%	420	92.9%	256	90.8%	0.001
	31-50 Years	104	7.3%	30	6.6%	16	5.7%	
	>50 years	12	0.8%	2	0.4%	10	3.5%	
Marital status	Unmarried	1182	83.0%	358	79.2%	224	79.4%	<0.001
	Married	242	17.0%	90	19.9%	52	18.4%	
	Divorced	0	0.0%	4	0.9%	6	2.1%	
Education	Primary	2	0.1%	2	0.4%	8	2.8%	<0.001
	Secondary	6	0.4%	4	0.9%	4	1.4%	
	Intermediate	132	9.3%	46	10.2%	28	9.9%	
	Bachelor	884	62.1%	272	60.2%	166	58.9%	
	Master or above	400	28.1%	128	28.3%	76	27.0%	
Occupation	Student	1028	72.2%	318	70.4%	204	72.3%	0.014
	Govt. Employee	94	6.6%	26	5.8%	14	5.0%	
	Non-Govt. Employee	140	9.8%	40	8.8%	16	5.7%	
	Businessman	30	2.1%	18	4.0%	12	4.3%	
	Unemployment	86	6.0%	30	6.6%	16	5.7%	
	Housewife	46	3.2%	20	4.4%	20	7.1%	
Profession	Not related to health	674	47.3%	294	65.0%	176	62.4%	<0.001
	Health related	750	52.7%	158	35.0%	106	37.6%	
Monthly income	<20,000	92	6.5%	54	11.9%	10	3.5%	<0.001
	20,000 to 40,000	122	8.6%	32	7.1%	24	8.5%	
	40,001 to 60,000	62	4.4%	24	5.3%	16	5.7%	
	>60,000	124	8.7%	22	4.9%	26	9.2%	
	No income	1024	71.9%	320	70.8%	206	73.0%	
Province	Punjab	666	46.8%	197	43.6%	126	44.7%	0.068
	Sindh	187	13.1%	54	11.9%	46	16.3%	
	KPK	205	14.4%	70	15.5%	36	12.8%	
	Baluchistan	155	10.9%	75	16.6%	33	11.7%	
	Gilgit-Baltistan	98	6.9%	25	5.5%	24	8.5%	
	Capital Islamabad	113	7.9%	31	6.9%	17	6.0%	
Residence	Rural	338	23.7%	144	31.9%	98	34.8%	<0.001
	Urban	1086	76.3%	308	68.1%	184	65.2%	

		M6. Do you believe that the COVID-19 vaccine can harm people's health as this has been developed in a very short period?						P-value
		No		Maybe		Yes		
		N	%	N	%	N	%	
Gender	Male	322	50.6%	492	54.1%	378	61.8%	<0.001
	Female	314	49.4%	418	45.9%	234	38.2%	
Age Groups	18-30 years	592	93.1%	838	92.1%	554	90.5%	0.022
	31-50 Years	40	6.3%	66	7.3%	44	7.2%	
	>50 years	4	0.6%	6	0.7%	14	2.3%	
Marital status	Unmarried	534	84.0%	742	81.5%	488	79.7%	0.059
	Married	102	16.0%	164	18.0%	118	19.3%	
	Divorced	0	0.0%	4	0.4%	6	1.0%	
Education	Primary	2	0.3%	2	0.2%	8	1.3%	0.291
	Secondary	4	0.6%	6	0.7%	4	0.7%	
	Intermediate	64	10.1%	82	9.0%	60	9.8%	
	Bachelor	390	61.3%	566	62.2%	366	59.8%	
	Master or above	176	27.7%	254	27.9%	174	28.4%	
Occupation	Student	450	70.8%	658	72.3%	442	72.2%	0.347
	Govt. Employee	36	5.7%	60	6.6%	38	6.2%	
	Non-Govt. Employee	62	9.7%	80	8.8%	54	8.8%	
	Businessman	14	2.2%	30	3.3%	16	2.6%	
	Unemployment	46	7.2%	56	6.2%	30	4.9%	
	Housewife	28	4.4%	26	2.9%	32	5.2%	
Profession	Not related to health	322	50.6%	502	55.2%	320	52.3%	0.195
	Health related	314	49.4%	408	44.8%	292	47.7%	
Monthly income	<20,000	54	8.5%	68	7.5%	34	5.6%	0.005
	20,000 to 40,000	52	8.2%	74	8.1%	52	8.5%	
	40,001 to 60,000	26	4.1%	48	5.3%	28	4.6%	
	>60,000	46	7.2%	54	5.9%	72	11.8%	
	No income	458	72.0%	666	73.2%	426	69.6%	
Province	Punjab	286	45.0%	417	45.8%	286	46.7%	0.371
	Sindh	80	12.6%	113	12.4%	94	15.4%	
	KPK	88	13.8%	139	15.3%	84	13.7%	
	Baluchistan	77	12.1%	112	12.3%	74	12.1%	
	Gilgit-Baltistan	51	8.0%	67	7.4%	29	4.7%	
	Capital Islamabad	54	8.5%	62	6.8%	45	7.4%	
Residence	Rural	182	28.6%	238	26.2%	160	26.1%	0.499
	Urban	454	71.4%	672	73.8%	452	73.9%	

		M7. Do you believe COVID-19 and its vaccine are non-Muslims' propaganda to rule the world?						P-value
		No		Maybe		Yes		
		N	%	N	%	N	%	
Gender	Male	644	52.3%	328	57.3%	220	62.1%	0.002
	Female	588	47.7%	244	42.7%	134	37.9%	
Age Groups	18-30 years	1120	90.9%	540	94.4%	324	91.5%	0.020
	31-50 Years	94	7.6%	32	5.6%	24	6.8%	
	>50 years	18	1.5%	0	0.0%	6	1.7%	
Marital status	Unmarried	1022	83.0%	456	79.7%	286	80.8%	0.001
	Married	210	17.0%	112	19.6%	62	17.5%	
	Divorced	0	0.0%	4	0.7%	6	1.7%	
Education	Primary	8	0.6%	2	0.3%	2	0.6%	0.036
	Secondary	6	0.5%	2	0.3%	6	1.7%	
	Intermediate	106	8.6%	68	11.9%	32	9.0%	
	Bachelor	744	60.4%	356	62.2%	222	62.7%	
	Master or above	368	29.9%	144	25.2%	92	26.0%	
Occupation	Student	860	69.8%	428	74.8%	262	74.0%	0.001
	Govt. Employee	80	6.5%	32	5.6%	22	6.2%	
	Non-Govt. Employee	138	11.2%	36	6.3%	22	6.2%	
	Businessman	30	2.4%	12	2.1%	18	5.1%	
	Unemployment	76	6.2%	42	7.3%	14	4.0%	
	Housewife	48	3.9%	22	3.8%	16	4.5%	
Profession	Not related to health	564	45.8%	372	65.0%	208	58.8%	<0.001
	Health related	668	54.2%	200	35.0%	146	41.2%	
Monthly income	<20,000	94	7.6%	46	8.0%	16	4.5%	<0.001
	20,000 to 40,000	118	9.6%	36	6.3%	24	6.8%	
	40,001 to 60,000	60	4.9%	18	3.1%	24	6.8%	
	>60,000	98	8.0%	32	5.6%	42	11.9%	
	No income	862	70.0%	440	76.9%	248	70.1%	
Province	Punjab	578	46.9%	258	45.1%	153	43.2%	0.155
	Sindh	166	13.5%	63	11.0%	58	16.4%	
	KPK	181	14.7%	85	14.9%	45	12.7%	
	Baluchistan	131	10.6%	82	14.3%	50	14.1%	
	Gilgit-Baltistan	79	6.4%	41	7.2%	27	7.6%	
	Capital Islamabad	97	7.9%	43	7.5%	21	5.9%	
Residence	Rural	306	24.8%	170	29.7%	104	29.4%	0.048
	Urban	926	75.2%	402	70.3%	250	70.6%	
